# Efficient CRISPR/Cas9-Mediated Gene Editing in an Interspecific Hybrid Poplar With a Highly Heterozygous Genome

**DOI:** 10.3389/fpls.2020.00996

**Published:** 2020-07-03

**Authors:** Jie Wang, Huaitong Wu, Yingnan Chen, Tongming Yin

**Affiliations:** Key Laboratory for Tree Breeding and Germplasm Improvement, Southern Modern Forestry Collaborative Innovation Center, College of Forestry, Nanjing Forestry University, Nanjing, China

**Keywords:** CRISPR/Cas9, *Populus davidiana × P. bolleana*, *PDS* gene, gene editing, single nucleotide polymorphism effect, off-target

## Abstract

Although the CRISPR/Cas9 system has been widely used for crop breeding, its application for the genetic improvement of trees has been limited, partly because of the outcrossing nature and substantial genomic heterozygosity of trees. Shanxin yang (*Populus davidiana* × *P. bolleana*), is a commercially important poplar clone that is widely grown in northern China. An established transformation protocol for this interspecific hybrid enables researchers to simultaneously investigate the efficiency and specificity of the CRISPR/Cas9-mediated manipulation of a highly heterozygous genome. Using the phytoene desaturase gene (*PDS*) as an example, we revealed that the CRISPR/Cas9 system could efficiently edit the Shanxin yang genome. Two sgRNAs were designed and incorporated into a single binary vector containing the Cas9 expression cassette. Among 62 independent transgenic lines, 85.5% exhibited an exclusively albino phenotype, revealing the total loss of PDS function. The Illumina sequencing results confirmed the targeted mutation of *PdbPDS* homologs induced by CRISPR/Cas9, and small insertions/deletions were the most common mutations. Biallelic and homozygous knockout mutations were detected at both target sites of the T_0_ transformants. Off-target activity was detected for sgRNA2 with a frequency of 3.2%. Additionally, the SNP interference of targeting specificity was assessed based on the sequence variation among *PdbPDS* homologs. A single mismatch at 19- or 10-bp from the PAM was tolerated by the CRISPR/Cas9 system. Therefore, multiple homologous genes were simultaneously edited despite the presence of a mismatch between the sgRNA and the target site. The establishment of a viable CRISPR/Cas9-based strategy for editing the Shanxin yang genome will not only accelerate the breeding process, but may also be relevant for other economically or scientifically important non-model plants species.

## Introduction

The CRISPR/Cas9 system is a revolutionary technology for plant breeding because of its simplicity, efficiency, and versatility (Bortesi and Fischer, 2015; Gao, 2018). This system consists of two essential components, the Cas9 nuclease and a single guide RNA (sgRNA), which can induce double-strand breaks at specific genomic sites (Jinek et al., 2012). The cellular repair of CRISPR/Cas9-mediated double-strand breaks through non-homologous end joining may result in gene modifications (Bortesi and Fischer, 2015). The ability to reprogram CRISPR/Cas9 to target any gene of interest using an engineered sgRNA has enabled plant breeders to develop new varieties with desired characteristics, including higher yields, improved quality, and increased resistance to disease and climate change. For example, CRISPR/Cas9-mediated inactivation of *OsGW5* in rice significantly increased the grain width and weight (Liu et al., 2017); low-gluten wheat was created by modifying the α-gliadin gene array (Sanchez-Leon et al., 2018); and the CRISPR-mediated genomic deletion of the *SlMlo1* locus generated tomato plants resistant to powdery mildew (Nekrasov et al., 2017).

Between 2013 and 2019, CRISPR/Cas9 technology has been successfully used to modify plant species from 45 genera across 24 families (Shan et al., 2020). However, the application of the CRISPR/Cas9 system to genetically alter woody perennials has been limited to only the following 10 species: apple (Nishitani et al., 2016), cacao (Fister et al., 2018), cassava (Odipio et al., 2017), citrus (Jia et al., 2017), coffee (Breitler et al., 2018), grape (Nakajima et al., 2017), kiwifruit (Wang et al., 2018), *Parasponia andersonii* (Van Zeijl et al., 2018), pomegranate (Chang et al., 2019), and poplar (Fan et al., 2015; Zhou et al., 2015). Recalcitrance to transformation and/or difficulties in regeneration are two predominant issues preventing the more widespread application of CRISPR in woody plants (Bewg et al., 2018). Moreover, their outcrossing nature and highly heterozygous genomes represent additional challenges to the genetic editing of tree species. The considerable abundance of single nucleotide polymorphisms (SNPs) in outcrossing species may restrict or even abolish CRISPR/Cas9-mediated genome editing (Jinek et al., 2012). In *Populus* species, one SNP altering the protospacer adjacent motif (PAM) site from NGG to NGA insulated the genome completely from editing (Zhou et al., 2015).

Shanxin yang (*Populus davidiana* × *P. bolleana*) is an elite aspen hybrid poplar bred for its cold and drought tolerance (Li et al., 2005). Because of its rapid growth, narrow crown, and stress resistance, Shanxin yang is an economically important cultivar that is widely grown in the northern provinces of China. Furthermore, an efficient transformation and regeneration system has been established for this hybrid poplar (Wang et al., 2011). Thus, many economically valuable traits can be improved *via* genetic modification. In this study, using the phytoene desaturase gene (*PDS*) as an example, we demonstrate the highly efficient editing of the Shanxin yang genome *via* a CRISPR/Cas9 system. Additionally, with the similar but not identical sequences of *PdbPDS* gene fragments, the effects of SNPs on genome editing efficiency were investigated. The establishment of a CRISPR/Cas9-based method for genetically manipulating in Shanxin yang may greatly facilitate the breeding of improved poplar cultivars. Moreover, the practical approach presented herein may be applicable for other valuable woody perennials that have highly heterozygous genomes but limited genomic resources.

## Materials and Methods

### Cloning of *PdbPDS* Fragments and Selection of CRISPR/Cas9 Target Sites

Genomic DNA was extracted from young leaves according to a modified CTAB (cetyltrimethyl ammonium bromide) method (Porebski et al., 1997). On the basis of the conserved sequence in two *Populus trichocarpa PDS* genes, primers F1 (5’-GTTGAATTTGGTTTTGGAGAA-3’) and R1 (5’- CATCTCTTGCTTCAAGCAATA-3’) were designed to amplify the 5’ end fragments of *PDS* genes in Shanxin yang. The PCR amplification was completed with KOD DNA polymerase (Toyobo, Japan) in a total volume of 25 μL, with the following reaction conditions: 94°C for 3 min; 32 cycles of 94°C for 30 s, 55°C for 30 s, and 72°C for 50 s; and 72°C for 10 min. The PCR products were purified with the AxyPrep™ DNA Gel Extraction Kit (Axygen Scientific, USA), cloned into the pEASY-Blunt vector (Transgen Biotech, China), and then analyzed by Sanger sequencing (Sangon Biotech, China). All possible Cas9 target sites within the obtained sequences were identified with the online software CRISPR-GE (http://skl.scau.edu.cn/, Xie et al., 2017). Two target sites were selected based on their genomic locations, GC contents, and potential off-target scores.

### Vector Construction and Plant Transformation

A gene-editing vector for *PdbPDS* expressing two sgRNAs was constructed as described by Xing et al. (2014). Briefly, the two target sites were incorporated into the following four primers: DT1-BsF: 5′-ATATATGGTCTCGATTG*TGAGTGCATTCAACTTGAGC*GTT-3′, 5′-DT1-F0: TG *TGAGTGCATTCAACTTGAGC*GTTTTAGAGCTAGAAATAGC-3′, DT2-R0: 5′-AAC *AGACCGGACCTTGATAACA*CAATCTCTTAGTCGACTCTAC-3′, and DT2-BsR: 5′-ATTATTGGTCTCGAAAC*AGACCGGACCTTGATAACA*CAA-3′. The italicized letters represent the target sequence. With these four primers, a PCR fragment (626 bp) was amplified from pCBC-DT1T2 and then incorporated between the *BsaI* sites of pHSE401 by Golden Gate cloning. The resulting pHSE401-2gR-PdbPDS recombinant plasmid was inserted into *Agrobacterium tumefaciens* (strain EHA105) cells according to a freeze–thaw method (Chen et al., 1994). The transformed clones with the correct sequence were identified by colony PCR and sequencing.

Shanxin yang seedlings were transformed with a slightly modified version of the method described by Wang et al. (2011). Specifically, leaf disks excised from aseptic seedlings were inoculated by swirling in the *A. tumefaciens* cultures for approximately 20 min. After the co-cultivation, the infected explants were washed several times with sterilized distilled water, and then pre-cultivated for one week on medium containing 200 mg/L cefotaxime and 150 mg/L timentin. The explants were then transferred to the shoot induction medium supplemented with 200 mg/L cefotaxime, 150 mg/L timentin, and 10mg/L hygromycin (Hyg). After the Hyg-resistant shoots were approximately 1.5 cm long, they were transferred to the rooting medium.

### Detection of Mutations

Genomic DNA was extracted from the leaves of each transgenic line and used as the template to amplify a *Hyg* gene (1,150 bp) with primers HygF (5′- CTACAAATCTATCTCTCTCG-3′) and HygR (5′- TATCTGGGAACTACTCACAC-3′) and the following PCR conditions: 94°C for 3 min; 35 cycles of 94°C for 30 s, 58°C for 30 s, 72°C for 70 s; and 72°C for 5 min. The PCR products were visualized on 1% agarose gels stained with GelRed (Generay, China). All of the *Hyg*-positive DNA samples as well as two wild-type (WT) DNA samples were used as templates to amplify the fragment spanning two target sites. The first round of PCR amplification was completed with primers F2 (5′-TTGGTTTTGGAGAAATGAGT-3′) and R2 (5′-GAAGAACGAAAGGATGAAGA-3′), and the following PCR conditions: 94°C for 3 min; 35 cycles of 94°C for 30 s, 58°C for 30 s, and 72°C for 30 s; and 72°C for 5 min. The amplification was verified by agarose gel electrophoresis. During the second round of PCR amplification, 12 barcoded forward primers and eight barcoded reverse primers were used to add a unique barcode to each sample. The PCR conditions were as follows: 98°C for 2 min; 30 cycles of 98°C for 10 s, 60°C for 15 s, and 72°C for 5 s; and 72°C for 5 min. Three indexed PCR-free libraries (each containing 32 samples) were then prepared with the TruSeq DNA Sample Preparation Kit (Illumina, USA). The libraries were sequenced with the Illumina HiSeq 2000 system.

Based on the barcode sequence, reads of each transgenic line were separated using Perl script. Raw reads were trimmed for quality using prinseq v0.20.4 (Schmieder and Edwards, 2011), and paired-reads were assembled using PANDAseq (Masella et al., 2012). After barcode trimming, representative sequences that have a frequency higher than 5% within a given sample were retained and aligned using DNAMAN v.8 (Lynnon Corporation).

## Results

### Detection of *PDS* Genes in Shanxin Yang and Selection of sgRNA Targets

**Figure 1:**
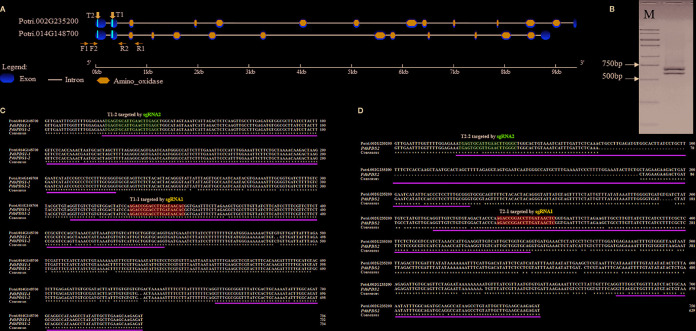
Targets locations and sequence alignments. **(A)** Structures of *PtPDS* genes in *P. trichocarpa*. T1 and T2 indicate the targets locations. F1 and R1 were used to amplify Shanxin yang *PDS* genes fragments. F2 and R2 were used to amplify the target region. **(B)** PCR amplification of *PdbPDS* genes fragments. **(C)** Alignment of two *PdbPDS1* allele fragments and Potri.014G148700. **(D)** Alignment of one *PdbPDS2* allele fragment and Potri.002G235200. In **(C**, **D)**, the green lines below aligned sequences indicate predicted exons within the amplified fragments; and the blue and pink boxes represent potential target sites that may be bind by sgRNA1 and sgRNA2, respectively. The first number in each target name represents the gene number, while the second number represents the number of sgRNA, e.g., T1-1 means the target site in *PdbPDS1* that can be bounded by sgRNA1.

The AtPDS3 protein sequence was used to identify two homologous genes (Potri.014G148700 and Potri.002G235200) in the *P. trichocarpa* genome (version 3.1, https://phytozome.jgi.doe.gov/pz/portal.html#!info?alias=Org_Ptrichocarpa_er). These genes shared a sequence identity of 92.01% at the amino acid level. The intra-genome synteny data in the Plant Genome Duplication Database (Lee et al., 2012) indicate these two *PtPDS* genes are paralogs that arose from segmental or whole-genome duplication events. To detect the *PDS* genes in Shanxin yang, a primer pair (F1 and R1) targeting a conserved sequence between the two *PtPDS* genes was designed to flank the first three exons (**Figure 1A**). Unexpectedly, two PCR products were amplified, one with the expected size of 730 bp, and another approximately 100 bp smaller (**Figure 1B**). Both amplified products were purified from agarose gels, cloned, and sequenced. A sequence alignment revealed that the longer product included two SNP-containing allelic fragments (*PdbPDS1-1* and *PdbPDS1-2*), which showed a higher similarity with Potri.014G148700 (**Figure 1C**). The shorter product comprised only one amplicon (*PdbPDS2*), which displayed a higher identity with Potri.002G235200. A comparison with Potri.002G235200 uncovered a 114 bp deletion in the first putative exon of *PdbPDS2* (**Figure 1D**). Because Shanxin yang is a diploid species, the unique *PdbPDS2* sequence suggests that the two alleles of this gene might be homozygous within the amplified fragment (*PdbPDS2-1* and *PdbPDS2-2*).

**Figure 2:**
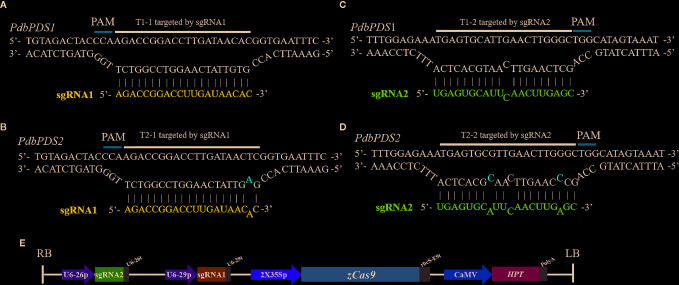
Schematic diagram of the complementarity between the sgRNAs and target sites, as well as the vector construction. **(A)** Perfect complementarity between sgRNA1 and T1-1. **(B)** Incomplete base-pairing between sgRNA1 and T2-1. **(C)** Incomplete base-pairing between sgRNA2 and T1-2. **(D)** Three mismatches in the putative off-target site T2-2. **(E)** T-DNA region of the constructed gene editing vector. The abbreviations are referred to Xing et al. (2014). U6-26p and U6-29p, two *Arabidopsis* U6 gene promoters; U6-26t and U6-29t, two U6-26 terminators with downstream sequence; 2x35Sp, 2x35S promoter; *zCas9*, *Zea mays* codon-optimized Cas9; rbcS-E9t, rbcS E9 terminator; CaMV, CaMV 35S promoter; *Hpt*, hygromycin phosphotransferase; PolyA, CaMV polyA signal. The sequence identity between 2x35Sp and CaMV is 94.55%.

On the basis of the allelic variation of *PdbPDS1*, two sgRNAs (sgRNA1 and sgRNA2) were designed to target two conserved sites located (**Figure 1A**). Candidate targets were also identified in *PdbPDS2*. The sgRNA1 might bind to T1-1 and T2-1, which were located in the second exons of two alleles of two genes, respectively (blue boxes in **Figures 1C, D**); and the sgRNA2 might bind to T1-2 and T2-2, which were located in the first exons of two alleles of two genes (pink boxes in **Figures 1C, D**). The sgRNA1 sequence perfectly matched the T1-1 (**Figure 2A**), while the T2-1 harbored a SNP 19 bp away from the PAM (**Figure 2B**). Therefore, the sgRNA1 had two perfectly matched sites (T1-1) and two single-mismatched sites (T2-1). To assess the SNP interference of sgRNA specificity at different positions, a mismatch was introduced into the sgRNA2 sequence (G replaced with C) 9 bp from the PAM (**Figure 2C**). Additionally, two naturally existing SNPs were detected in the corresponding site of *PdbPDS2* (T2-2, **Figure 2D**). Thus, the sgRNA2 had two imperfect target sites (T1-2, 1 bp mismatch) and two potential off-target sites (T2-2, 3 bp mismatch). A single binary vector containing two sgRNA expression cassettes was constructed, in which sgRNA1 and 2 were expressed under the control of U6-29P and U6-26P, respectively (**Figure 2E**).

### Phenotypes of CRISPR/Cas9 Transgenic Poplar

**Figure 3:**
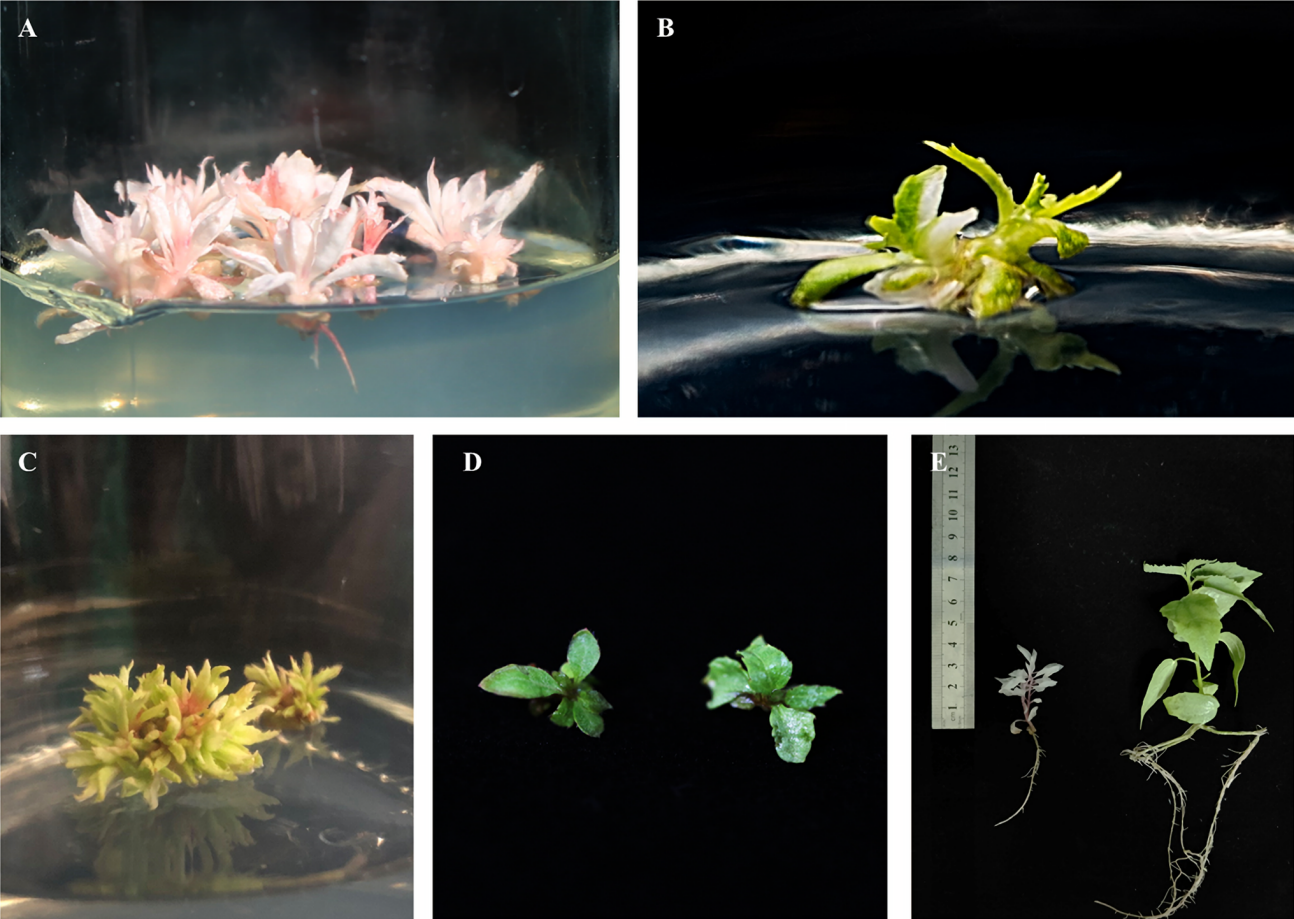
Representative Shanxin yang phenotypes resulting from *PDS* gene mutations. Total albino **(A)**, variegated **(B)**, and pale green **(C)** phenotypes of the regenerated shoots. **(D)** Wild-type (WT) Shanxin yang shoots. **(E)** Comparison between the 1-month-old albino and WT seedlings.

The pHSE401-2gR-PdbPDS recombinant plasmid containing the *Cas9* expression cassette, two sgRNA cassettes, and the hygromycin phosphotransferase (*HPT*) gene (selection marker) was transformed into Shanxin yang leaf disks *via* Agrobacterium-mediated transformation. Approximately two months later, Hyg-resistant shoot buds were regenerated with the following four phenotypes: total albino (**Figure 3A**), variegated with both albino and green leaves (**Figure 3B**), pale green (**Figure 3C**), or normal green (i.e., same as the WT) (**Figure 3D**). The regenerated buds were transferred to the selective rooting medium. Finally, we obtained 84 putative transgenic plantlets from 62 independent lines (**Table 1**). A PCR analysis revealed that the *HPT* gene was present in all of the T_0_ seedlings (**Figure S1**).

**Table 1:** Summary of the phenotypes of the T_0_ transgenic plants.

Phenotype	No. of lines	No. of plantlets
Albino	53	77
Mosaic	1	1
Pale green or yellow	2	2
Green	6	6
Total	62	86

Among the 62 transgenic lines, 53 (85.5%) exhibited an exclusively albino phenotype, reflecting the total loss of PDS function. Additionally, one line exhibited a mosaic phenotype, whereas two lines were pale green or yellow (**Table 1**). The remaining six lines were phenotypically the same as the WT control. The albino plantlets were weak and small (**Figure 3E**), and died during acclimatization.

### Characterization of the Targeted Editing of the *PdbPDS* Genes

With the F2/R2 primer pair, DNA segments spanning the two targets were amplified for 62 transgenic plants representing the 62 different lines (**Table 1**). Each PCR product comprised three different fragments, which were derived from *PdbPDS1-1*, *PdbPDS1-2,* and *PdbPDS2*. Amplicons of each sample were uniquely barcoded and subjected to Illumina sequencing. Mutations to at least one target site were detected in 60 of the 62 analyzed plants (i.e., 96.8%) (**Table S1**). The transgenic plants exhibited an albino phenotype when more than two alleles of the two genes was edited, whereas they were pale green or normal green when only one gene was mutated (**Table 2**). The mosaic plant contained chimeric mutations, in which five distinct *PdbPDS1* alleles were detected (**Table S1**). Moreover, chimeric mutations were also identified in 13 albino plants, each of which had at least three different *PdbPDS1* alleles (**Table 2** and **Table S1**). Therefore, chimerism occurred in the transformants at a rate of about 23.3%.

**Table 2:** Summary of the edited genes and phenotypes of the sequenced transgenic plants.

		Edited or not									
Gene name	*PdbPDS1-1*	Y	Y	Y	N	Y	Y	N	Y	N	N
	*PdbPDS1-2*	Y	Y	Y	Y	Y/N	N	N	N	Y	N
	*PdbPDS2-1*	Y	Y	N	Y	N	N	Y	N	N	N
	*PdbPDS2-2*	Y	N	N	Y	N	N	N	N	N	N
Phenotype		Albino	Albino	Albino	Albino	Mosaic	Pale green	Pale green	Green	Green	Green
No. of sequenced plants		34	7	11	1	1	1	1	3	1	2
No. of plants having chimeric mutation		9	3	1	0	1	0	0	0	0	0

Y and N represents the gene is edited or not, respectively. PdbPDS1 possesses two heterozygous alleles (PdbPDS1-1 and PdbPDS1-2), while PdbPDS2 possesses two homologous alleles (PdbPDS2-1 and PdbPDS2-2).

**Figure 4:**
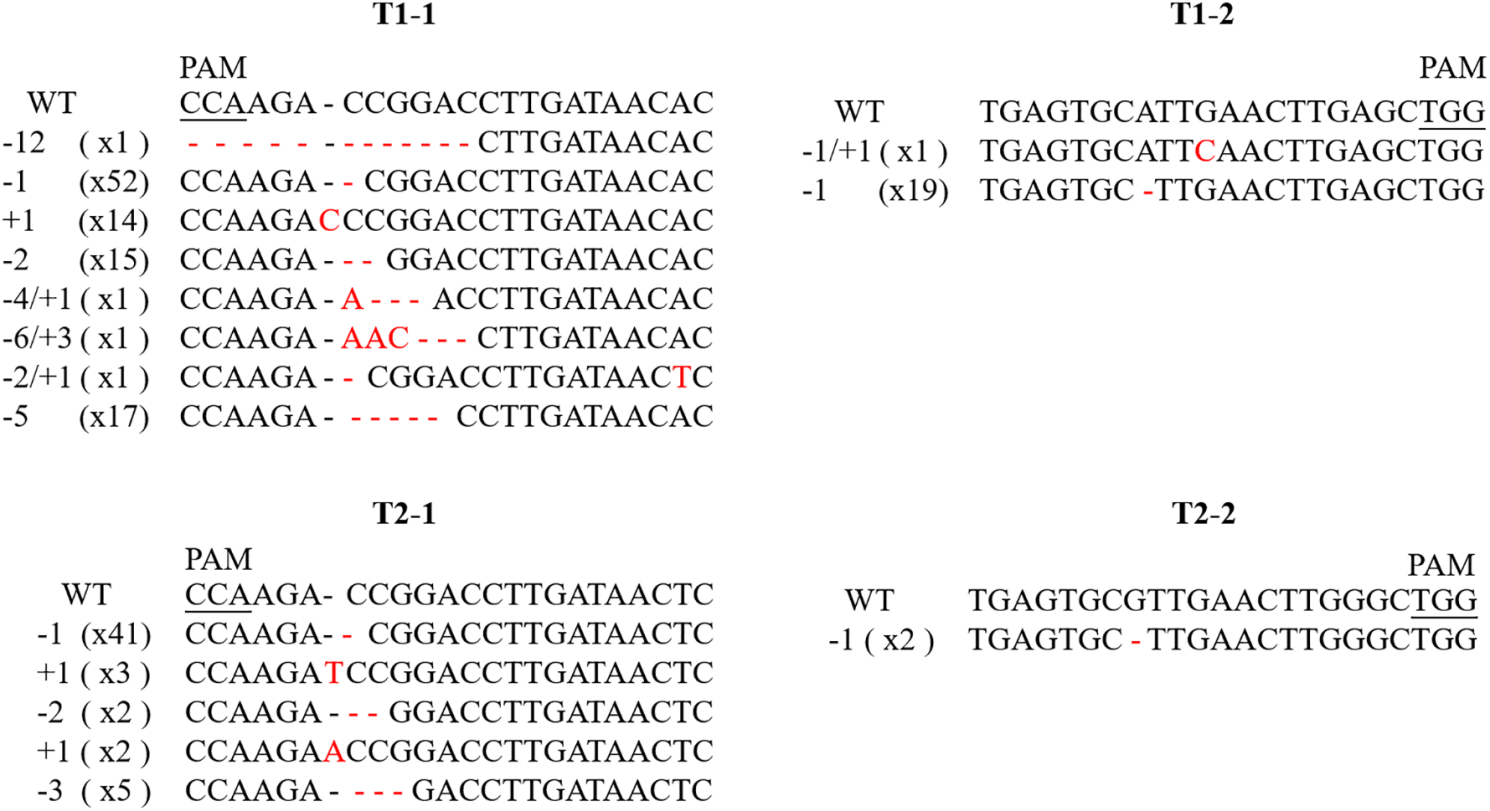
Targeted mutations of *PdbPDS* genes induced by CRISPR/Cas9. The top row of each panel is the WT sequence of the given sgRNA, and the protospacer adjacent motif (PAM) is underlined. Deletions are indicated by red dashes. Insertions and substitutions are indicated by red letters. The number in brackets represents the number of plants with the specific mutant allele.

A total of 119 gene-editing events were identified in 60 plants with *PDS* mutations (**Table S1**). The mutation type and mutagenesis rate were analyzed in detail for every target site. Small insertions/deletions (indels) were prevalent among the editing events (**Table 3**), with eight, five, and two different types of indels in T1-1, T2-1, and T1-2, respectively (**Figure 4**). The analyses of T1-1 and T1-2 in *PdbPDS1* revealed mutations in at least one allele in 55 and 20 plants, respectively (**Table 3**). Of the 55 plants, 42 had mutations in both alleles (76.4%), including nine homozygous mutations and 33 biallelic mutations (**Table 3**), whereas among the 20 plants, only one had mutations at T1-2 in both alleles (**Table 3**). Notably, 12 plants with modifications to both T1-1 and T1-2 in *PdbPDS1-1* and four plants with four altered targets in two *PdbPDS1* alleles were identified (**Table S1**), but no large deletion or insertion was detected, suggesting that Cas9 cleaved the two targets in one gene sequentially rather than simultaneously. Regarding the analyses of T2-1 in *PdbPDS2*, mutations in at least one allele were detected in 45 plants, of which 34 had mutations in both alleles (75.6%), including 25 homozygous mutations and nine biallelic mutations (**Table 3**).

**Table 3:** Summary of the mutation types at each target site.

Mutation type	No. of plants			
	Targeted by sgRNA1		Targeted by sgRNA2	
	T1-1	T2-1	T1-2	T2-2
Insertion only	0	1	0	0
Deletion only	40	40	19	2
Insertion and deletion	15	4	0	0
Subsitution	0	0	1	0
Homozygous mutation	9	25	1	0
Heterozygous mutation	3	9	19	2
Biallelic mutation	33	9	0	0
Chimeric mutation	10	2	0	0
Plants with mutation	55	45	20	2
Unmodified	7	17	42	60
Mutation rate	88.7%	72.5%	32.3%	3.2%

The sgRNA1 was intended to two alleles of two *PdbPDS* genes (T1-1 and T2-1). Among the 46 plants containing mutations introduced by sgRNA1, 42 (i.e., nearly 90%) had mutations in more than one gene and 27 (i.e., 43.5%) had mutations in all four genes (**Table S2**). Moreover, the editing rate of sgRNA1 at T1-1 was 88.7%, but at T2-1, the rate decreased to 72.5% (**Table 3**) because of the presence of a SNP in the T2-1 sequence (**Figure 2B**). Although the single-base mismatch was distal to the PAM site, it significantly decreased the editing efficiency of sgRNA1 (P < 0.05, χ^2^ test). Compared with the high efficiency of sgRNA1, the editing rate of sgRNA2 at T1-2 was only 32.2%, which can be partly explained by the mismatch in the middle of the target site (**Figure 2C**) as well as the relatively low efficiency of sgRNA2 itself. Additionally, T2-2 was predicted to be a potential off-target site, harboring three mismatches (**Figure 2D**). Among the 62 sequenced plants, two had mutations at this site (i.e., 3.2%) (**Figure 4**).

## Discussion

Shanxin yang is a typical interspecific outcrossing woody perennial species with a highly heterozygous genome. Using a pair of conserved primers, we amplified three different *PDS* genes sequences, including two haplotypes of *PdbPDS1* and one haplotype of *PdbPDS2*. *PdbPDS1* is a heterozygous locus, while *PdbPDS2* can be either hemizygous or homozygous. If *PdbPDS2* only presents on one of the chromatids (hemizygous), the wild-type allele cannot be detected in the *PdbPDS2* edited plants unless the edited line is a chimeric plant. We obtained nine lines showing heterozygous mutations in *PdbPDS2*, i.e., both the edited and unedited *PdbPDS2* were identified in these lines (**Table 3**). In the nine *PdbPDS2* edited lines, *PdbPDS1* was edited simultaneously. Based on the editing results of *PdbPDS1*, seven of the nine edited plants are not chimeras. Therefore, *PdbPDS2* should possess two homologous alleles in Shanxin yang. According to the gene editing results (**Table 2**), *PdbPDS1* and *PdbPDS2* may be both functional, and they regulate the chlorophyll biosynthesis in a dose-dependent manner. If more than two alleles of the two genes were edited, the transgenic plants appeared albino.

Two sgRNAs were designed to evaluate the efficiency and specificity of the CRISPR/Cas9-mediated genomic mutations in Shanxin yang. Our data revealed that sgRNA1 was able to simultaneously edit multiple genes, and 43.5% of the transgenic plants had mutations at all four target sites (**Table S2**). Multigene editing *via* CRISPR/Cas9 guided by a single sgRNA has been reported for many plant species (Lawrenson et al., 2015; Braatz et al., 2017; Gao et al., 2017; Yu et al., 2018), but the efficiencies of the multigene mutations were not always provided. In a previous study of tetraploid potato (*Solanum tuberosum*), only 2% of the regenerated lines had mutations in all four alleles (Andersson et al., 2017). Additionally, Zhang et al. (2016) reported that 10% of 80 T_0_ mutants had all six alleles simultaneously knocked out in hexaploid wheat (*Triticum aestivum* L.). Compared with strategies involving the expression of multiple sgRNAs for multiplex genome editing (Minkenberg et al., 2017), the use of a single sgRNA has the advantage of minimizing the risk of off-target mutations (Yu et al., 2018).

Notably, sgRNA1 is identical to the target region in *PdbPDS1*, whereas it differs from the target in *PdbPDS2* because of a SNP 19bp from the PAM. However, homozygous and biallelic mutations in *PdbPDS2* were still frequently detected (**Table 3**). This observation is consistent with the results of earlier studies in which genes with highly similar but not identical sequences were edited simultaneously by a single minimally stringent sgRNA (Lawrenson et al., 2015; Endo et al., 2016; Andersson et al., 2017; Braatz et al., 2017; Gao et al., 2017; Kanazashi et al., 2018). For example, in rice (*Oryza sativa*), three *CDK* genes can be mutated by one sgRNA even with an imperfect target match (Endo et al., 2016). In allotetraploid cotton (*Gossypium hirsutum* L.), an sgRNA with a mismatch 12 bp upstream of the PAM reportedly can mutate in all four *GhCLA1* homoeoalleles (Gao et al., 2017). Moreover, the mutation of three or four closely related genes with one incompletely matched sgRNA has been reported for *Brassica oleracea* and soybean (*Glycine max*) (Lawrenson et al., 2015; Kanazashi et al., 2018). These results suggest that although site-specific mutagenesis is highly desirable and necessary for characterizing gene functions, the tolerance of mismatches (as few as one or two nucleotide differences) in the target region can be exploited for multi-homologous or multi-paralogous gene editing, especially for polyploid plants.

The 8–12 nucleotides sequence upstream of the PAM is the seed region critical for target affinity and specificity (Semenova et al., 2011; Wiedenheft et al., 2011; Belhaj et al., 2015). Previous studies proved that a single-base mismatch within the 12 bp adjacent to the PAM can prevent the genomic cleavage by Cas9 (Sapranauskas et al., 2011; Jinek et al., 2012; Cong et al., 2013). In the current study, the mismatch in sgRNA2 was 10 bp from the PAM, but 32.2% of the transgenic plants had mutations at the target site (T1-2). This result is in accordance with the findings of Hinz et al. (2016), who demonstrated that a mismatch at a position +10 nucleotides from the PAM minimally affects Cas9 activity *in vitro*. Similarly, in another study, the sgRNA targeting four *BnALC* alleles in oilseed rape (*Brassica napus*) contained a SNP 10 nucleotides upstream of the PAM (Braatz et al., 2017). In contrast, the inhibitory effect of a single SNP on gene editing efficiency was observed in an earlier investigation of poplar [*P. tremula* × *alba* (717-1B4)], in which one SNP near or within the PAM prevented the Cas9-mediated cleavage (Zhou et al., 2015). These results support the previous finding that a SNP located distally from the PAM often has a limited effect on Cas9 activity (Jinek et al., 2012; Pattanayak et al., 2013). However, these studies collectively suggest that the length of the seed region varies among different systems, and more work is needed to elucidate the effects of SNPs on CRISPR/Cas9-mediated genome editing.

Off-target activity is another major concern of the CRISPR/Cas9 system. In this study, T2-2 was a putative off-target site of sgRNA2 in *PbdPDS2*. Although T2-2 has three mismatches with the sgRNAs, of the 62 transgenic plants, two (3.2%) had off-target mutations at this site. A high-frequency of off-target mutations has been reported for human cells and mouse embryos (Fu et al., 2013; Aryal et al., 2018). In plants, off-target mutagenesis was rare in some studies (Bilichak and Eudes, 2016; Wolt et al., 2016; Li et al., 2019), but was detected at frequencies of 1.6–13% in other studies (Upadhyay et al., 2013; Zhang et al., 2014; Jacobs et al., 2015; Sauer et al., 2016). The potential high frequency of off-target mutations in *Arabidopsis thaliana* was recently revealed by Zhang et al. (2018). Moreover, the frequency of off-target mutations apparently increases in the T2 progeny (Zhang et al., 2018). Therefore, special attention should be paid to limit the risk of off-target mutation, especially for perennial plants, such as trees, since the CRISPR/Cas9 system may function for many months or even years in these plant species.

## Data Availability Statement

All datasets generated for this study are included in the article/**Supplementary Material**.

## Author Contributions

JW performed the experiments and prepared the manuscript. HW helped in analyzing the sequencing data. YC and TY designed the study and helped to draft the manuscript. All authors contributed to the article and approved the submitted version.

## Conflict of Interest

The authors declare that the research was conducted in the absence of any commercial or financial relationships that could be construed as a potential conflict of interest.
