# Supplementary figures and images for: Efficient CRISPR/Cas9-Mediated Gene Editing in an Interspecific Hybrid Poplar With a Highly Heterozygous Genome
Source: Front Plant Sci. 2020 Jul 3;11:996. doi: 10.3389/fpls.2020.00996 (PMC7347981; doi:10.3389/fpls.2020.00996)

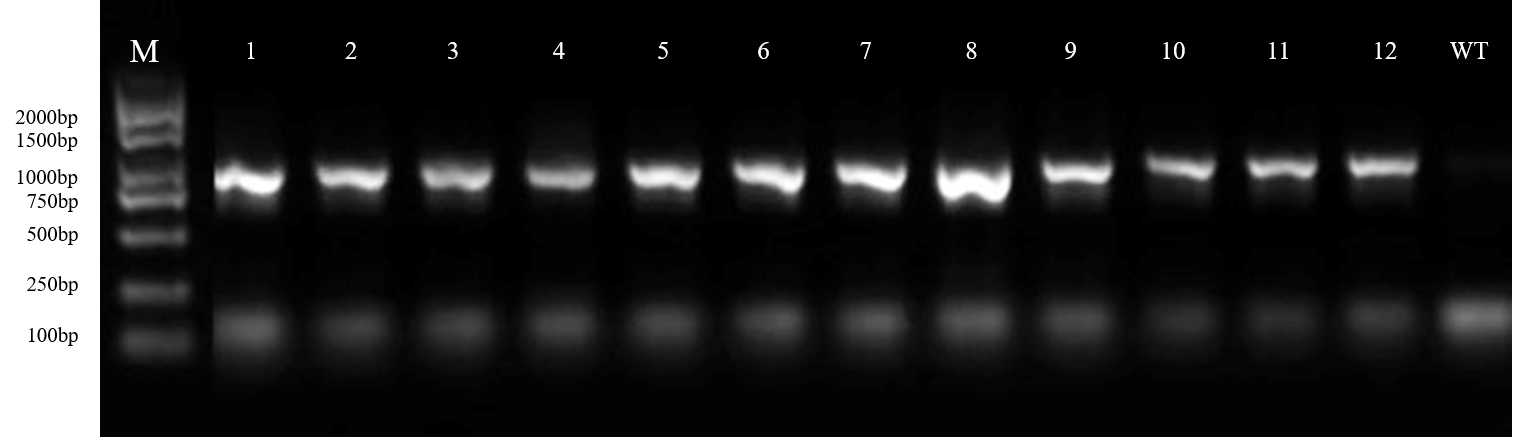

Supplement: Supplementary file 1 [file Image_1.tif]
