# Supplementary material for: Efficient CRISPR/Cas9-Mediated Gene Editing in an Interspecific Hybrid Poplar With a Highly Heterozygous Genome
Source: Front Plant Sci. 2020 Jul 3;11:996. doi: 10.3389/fpls.2020.00996 (PMC7347981; doi:10.3389/fpls.2020.00996)
Supplement: Supplementary file 3 [file Table_2.docx]

Table S2 Numbers and rates of plants containing one to four mutated sgRNA1 target sites

|  | Editing in only one site | Editing in two sites | Editing in three sites | Editing in four sites |
| --- | --- | --- | --- | --- |
| Plant ID | #27, #32, #44, #72, #86 | #37, #55, #42, #90, #43  #96, #47, #52 | #2, #92, #3, #26, #45, #60 | #4, #23, #41, #73, #83, #94  #8, #25, #56, #75, #87, #95  #11, #30, #58, #78, #89, #18  #35, #69, #79, #91, #22, #38  #70, #80,#93 |
| Total | 5 | 8 | 6 | 27 |
| Rate | 8.06% | 12.9% | 9.67% | 43.5% |

Note: the rate was calculated by dividing the no. of plants with given mutant site(s) by the total no. of transgenic plants.
